# Tracking of unpredictable moving stimuli by pigeons

**DOI:** 10.3758/s13420-020-00412-x

**Published:** 2020-02-03

**Authors:** Anna Wilkinson, Kimberly Kirkpatrick

**Affiliations:** 1 Animal Behaviour, Cognition and Welfare Group, School of Life Sciences, University of Lincoln, Lincoln, LN6 7DL UK; 2 Department of Psychology, University of York, York, UK; 3 Department of Psychological Sciences, Kansas State University, Manhattan, KS USA

**Keywords:** motion perception, visual perception, tracking, anticipation, pigeon

## Abstract

Despite being observed throughout the animal kingdom, catching a moving object is a complex task and little is known about the mechanisms that underlie this behavior in non-human animals. Three experiments examined the role of prediction in capture of a moving object by pigeons. In Experiment 1, a stimulus moved in a linear trajectory, but sometimes made an unexpected 90^o^ turn. The sudden turn had only a modest effect on capture and error location, and the analyses suggested that the birds had adjusted their tracking to the novel motion. In Experiment 2, the role of visual input during a turn was tested by inserting disappearances (either 1.5 cm or 4.5 cm) on both the straight and turn trials. The addition of the disappearance had little effect on capture success, but delayed capture location with the larger disappearance leading to greater delay. Error analyses indicated that the birds adapted to the post-turn, post-disappearance motion. Experiment 3 tested the role of visual input when the motion disappeared behind an occluder and emerged in either a straight line or at a 90^o^ angle. The occluder produced a disruption in capture success but did not delay capture. Error analyses indicated that the birds did not adjust their tracking to the new motion on turn trials following occlusion. The combined results indicate that pigeons can anticipate the future position of a stimulus, and can adapt to sudden, unpredictable changes in motion but do so better after a disappearance than after an occlusion.

Successful capture of a moving object is a complex task. It requires the organism to perceive the object, determine its path of movement, plan the object-directed action and move the capturing device (e.g. claw, hand or beak) to the correct location for interception (Von Hofsten, 1987). Despite the sophisticated nature of tracking and/or capture, these abilities can be seen throughout the animal kingdom in predator-prey interactions, mating displays and shoaling/flocking behavior. Furthermore, object tracking has been experimentally observed in many classes of non-human animals in both field and laboratory settings including fish (Lanchester & Mark, 1975), reptiles (Burger, Gochfield, & Murray, 1992), birds (Neiworth & Rilling, 1987; Rilling, LaClaire, & Warner, 1993; Ristau, 1991a, 1991b; Shifferman & Eilam, 2004) and mammals (Byers, 2002; Shaffer, Krachunas, Eddy, & McBeath, 2004). However, little experimental research with non-human animals has examined the nature of these abilities, and the underlying mechanisms are poorly understood.

The present set of experiments investigated the role of prediction in tracking and capture of a moving stimulus in the pigeon. Much is known about how pigeons perceive static images (e.g., Aust, 2017; Cook, 2001; Fagot, 2000; Lea et al., 2018; Stephan, Wilkinson, & Huber, 2013). However, only a handful of investigations have assessed how they perceive motion. There is evidence to suggest that pigeons perceive apparent motion as real (Siegel, 1970); they can also discriminate between moving and stationary objects (Hodos, Smith, & Bonbright, 1976), represent movement when it is out of sight (Neiworth & Rilling, 1987), and form motion categories (Dittrich & Lea, 1993; Lea & Dittrich, 2000). Cook and Katz (1999) have even suggested that the presence of motion allows superior classification of novel angles of 3D objects. However, motion does not aid all discriminations (Daniel & Katz, 2016).

A small number of studies have examined tracking behavior in pigeons (Lea, Chow, Meier, McLaren, & Verbruggen, 2019; Pisacreta, 1982; Rilling, 1992; Rilling & LaClaire, 1989; Wilkinson & Kirkpatrick, 2009, 2010). Rilling and LaClaire (1989) showed that pigeons are able to track and capture a moving object. Using a complex motion that travelled at a sinusoidal velocity and direction on a video monitor, they found that the pigeons could successfully intercept the stimulus by pecking at a touch screen but tended to chase the stimulus. Interestingly, the errors were a constant temporal amount behind the stimulus, suggesting that the pigeons were fixating on the current object location and then pecking in that location without attempting to anticipate ahead of the current position. Later experiments (Rilling, 1992) again found a similar pattern of lagging behavior. Recently, Lea et al. (2019) revealed that pigeons were able to adapt to unexpected changes in trajectory with differences in error latencies being observed after a change in direction.

Wilkinson and Kirkpatrick (2009) presented pigeons with a moving stimulus that was systematically varied in terms of size and velocity. Both manipulations affected the birds’ pecking behavior but in different ways; the size of the stimulus controlled capture success whereas the speed controlled error position. Two factors were identified as key elements in the tracking process. The first was lag time, which is a multiplicative factor. It most likely comprises of the time taken by the pigeon to fixate on the stimulus and land a peck on the touch screen. The second factor was an additive factor, peck bias, which allowed the birds to (partially) compensate for the lag time. The results suggested that the birds were using a spatial rather than temporal bias to anticipate ahead of the stimulus. However, it is unclear how, and to what extent the birds used the peck bias to anticipate ahead of the stimulus position. An additional means of testing predictive extrapolation is using sudden unpredictable perturbations in motion. This method can reveal more information about the nature of the anticipatory process.

Very little is known about the role of prediction in motion tracking by non-human animals, however this has been extensively studied in human infants (Gredebaak, von Hofsten, & Boudreau, 2002; von Hofsten, Feng, & Spelke, 2000; von Hofsten & Rosander, 2018; von Hofsten, Vishton, Spelke, Feng, & Rosander, 1998) and adults (Sanderson & Whiting, 1974; Sharpe & Whiting, 1974; Whiting, 1969; Whiting, Gill, & Stephenson, 1970). Von Hofsten et al. (1998) found that infants can anticipate motion 200 ms ahead of the current object position. Their predictive reaches appeared to be based on linear components of the motion. The authors suggested that infants possess a predictive extrapolation mechanism that uses physical constraints, such as rules of inertia, to predict the future position of a target. When presented with sudden changes in trajectory, the infants were unable to learn to predict the non-inertial motion even when they received the same non-linear motion for six trials in a row. This suggests that planning of predictive actions is affected by the observed object motion early in the trial and not by the remembered trajectory from previous trials. Thus, it seems that the visual input provided by the motion at the beginning of the trial may override the infant’s ability to use the knowledge they acquired during previous experience with non-linear trials.

Similarly, adult humans appear to predict approximately 200 ms ahead of the object motion. Whiting et al. (1970) found that adults benefited from being allowed time to process trajectory information in a ball interception task. The greater amount of time available to process information about velocity and direction, the more accurate the prediction of the balls’ future position. This finding was even consistent over short occlusions (Sharpe & Whiting, 1974). Interestingly, when the occlusion lasted for more than 80 ms the advantage seen in the time-to-prepare effect decreased and entirely disappeared when an occlusion lasted 240 ms. These findings suggest that adults are unable to anticipate the motion over the 240 ms occlusion and provide further evidence that humans extrapolate approximately 200 ms ahead of the object motion.

## Experiment 1

The aim of this experiment was to examine whether pigeons could adapt to sudden, unpredictable changes in stimulus trajectory. Our previous study (Wilkinson & Kirkpatrick, 2009) revealed the importance of long-term tracking history effects on how the birds tracked a novel motion, particularly in terms of peck bias. This experiment examined the role of within-trial history effects when the motion path changed suddenly during a trial. It is possible that, like the infants, a sudden change in direction may reveal a linear extrapolation process in the pigeon.

### Method

#### Animals

Three captive-bred pigeons (*Columba livia*) served as the experimental subjects: Black 50 (B50), Violet 42 (V42), and Green 83 (G83). They had recently participated in experiments where they captured simple linear motions (Wilkinson & Kirkpatrick, 2009), but they had no experience with sudden changes in trajectory.

The birds were housed in individual cages in a colony room on a 12:12 light-dark cycle with light onset at 8 a.m. Each bird was maintained at 85-90% of its free-feeding weight by the delivery of individual Noyes pigeon pellets in the experimental apparatus and supplementary access to grain in the home cage, ranging from 5-20 g per day. They were allowed free access to grit and water in the home cages. The birds were placed in a flight cage over the weekend to receive exercise and a bath; they did not participate in the experiment while they were in the flight cage. While in the flight cage, the birds received free access to grit and water and a once daily feeding of grain in the amount of 20 g per bird, scattered among the bedding at the bottom of the cage. All procedures were approved by the Ethics Board at the University of York.

#### Apparatus

The pigeons were trained and tested in two 35 × 32 × 24 cm operant chambers housed inside of a sound- and light-attenuating box (Med Associates). One wall of the chamber was fitted with an 18 × 25 cm resistive touch screen (Elotouch Systems, Accutouch) that was situated in front of a 15-in TFT monitor that was turned on its side. The monitor was set at a resolution of 640 × 480 pixels for the duration of the experiment. On the opposite wall of the chamber was a magazine pellet dispenser (Med Associates, ENV-203) and clicker (Med Associates, ENV-135M). Individual 45-mg pigeon pellets were delivered through a rubber tube into a food cup (Med Associates, ENV-200-R1M) that was located 2 cm above the grid floor. A houselight was located on the top-right corner of the wall above the food cup; this delivered diffuse illumination to the pigeon chamber at an intensity of approximately 200 lux (Med Associates, ENV-227M). A speaker, which was positioned outside the pigeon chambers, emitted a diffuse 60-dB white noise to mask sounds outside of the room.

Responses were recorded from the touch screen via a USB touch screen controller (Elotouch Systems, 3000U USB controller). Control of the feeder and houselight was accomplished by a digital I/O card (National Instruments, PCI-6503). A video splitter (Rextron, BSA12) allowed simultaneous presentation of images to the control room and operant chamber. Two Viglen Genie P4 computers located in an adjacent room delivered the experimental procedures and recorded data in E-prime v1.1. At the time of each peck, the location of the peck and the position of the stimulus were recorded in the form of XY coordinates with a time tag.

#### Procedure

##### Training

Because the pigeons had recent experience capturing linear motions (Wilkinson & Kirkpatrick, 2009), they received only a brief phase of initial training. The stimulus was a yellow circle that measured 0.55 cm and traveled at 3.40 cm/s. It could appear from one of five positions along any of the four sides of the touch screen. Both the leftward and rightward motions could appear along the vertical edge of the screen at 10, 12.5, 15, 17.5, or 20 cm displacement from the top of the screen. The upward and downward motions could appear along the horizontal edge of the screen at 7.5, 10.0, 12.5, 15.0, or 17.5 cm displacement from the left side of the screen. In all cases, the stimulus moved along a linear path directly towards the opposite side of the screen. There were a total of 20 trial types (4 motions × 5 starting positions) and these were randomly intermixed within a session; the birds received 3 trials of each type within a session for a total of 60 trials.

If a peck occurred anywhere within the stimulus boundary it counted as a catch, the screen darkened and the pigeon was rewarded with three food pellets. If the bird did not successfully capture the stimulus, it moved smoothly off the screen when it reached the opposite side. In this case, the bird was not rewarded and instead the intertrial interval began. Each training session consisted of 60 trials that were separated by an intertrial interval of 5 s, during which the screen was dark. The birds were trained to an acquisition criterion of two consecutive sessions with at least 70% of trials ending in a capture response. All three birds reached criterion within two sessions, due to their prior experience with the training task.

##### Testing

Following the training phase, the birds received a test during which a random subset of the trials delivered a motion with a sudden change in direction. These turn trials were presented in the same manner as the training trials with the following exceptions: (1) the stimulus travelled in a linear path for a minimum of 7.5 cm and a maximum of 15.0 cm before making a sudden 90° turn; (2) during the pre-turn motion, any pecks that fell on the stimulus had no consequence; (3) following the turn, the normal training contingencies were in place. Pecks on the stimulus were counted as captures and resulted in termination of the trial and food delivery. Misses resulted in the stimulus continuing towards the screen boundary in a linear path. If the pigeon failed to capture the stimulus, it would move smoothly off screen when it reached the opposite side.

There were eight types of turn trials, labeled according to pre- and post-turn motion direction: Right-Down, Left-Down, Right-Up, Left-Up, Down-Right, Up-Right, Down-Left, and Up-Left. There were three presentations of each of the turn trials, which were randomly intermixed among the 60 original training trials, yielding a total of 84 trials. In all other respects, testing was administered in the same manner as original training.

#### Data Analysis

Capture responses were assessed with two measures. The proportion of trials ending in a capture was the number of trials ending in a capture divided by the total number of trials in a session. The distance travelled before capture was computed from the point where the stimulus first became eligible for capture to the point of capture. Analyses on turn trials were conducted on pecks that occurred after the turn, once the stimulus was eligible for capture. Because the capture area on turn trials was less than on training trials, the data from training trials were truncated to equate capture opportunity on the two trial types. This was accomplished by only including captures that occurred within the initial portion of the straight trial that was equal in distance to the comparable turn trials (e.g., right-down turn trials were equated to the initial path on down straight trials); captures occurring beyond this initial area were excluded from the analysis.

For the error analysis, anticipatory and lagging errors were examined by determining the error position relative to the center of the stimulus. Errors in the same dimension as the motion (motion-relevant errors) were expressed as lagging behind the stimulus or leading in front of the stimulus.

In addition, to examine within-trial history effects on errors, a regression analysis was conducted. It assessed the extent to which the previous and current motion directions predicted the error positions after the turn or following trial onset on straight trials. The previous motion was coded as the initial motion on both turn and straight trials, and the current motion was coded as the post-turn motion on turn trials and as the initial motion on straight trials. For example, on a rightward motion straight trial, the previous motion was rightward and the current motion was rightward, whereas on a rightward-upward turn trial the initial motion was rightward and the current motion was upward. The errors here were entered as error positions which coded the distance of the peck from the center of the stimulus in both the X and Y planes.

For all analyses of turn trials, except the regressions, the data was collapsed across motion direction; this was for two reasons. First, although different motions resulted in somewhat different pecking patterns in the straight portion of the trials, the effect of the turn was highly consistent across motion types. Second, due to the small number of turn tests per session the data on turn trials were more variable than on straight trials. By collapsing across motion types, this resulted in an increase in stability of the test data.

### Results

#### Capture Responses

**Fig. 1.**
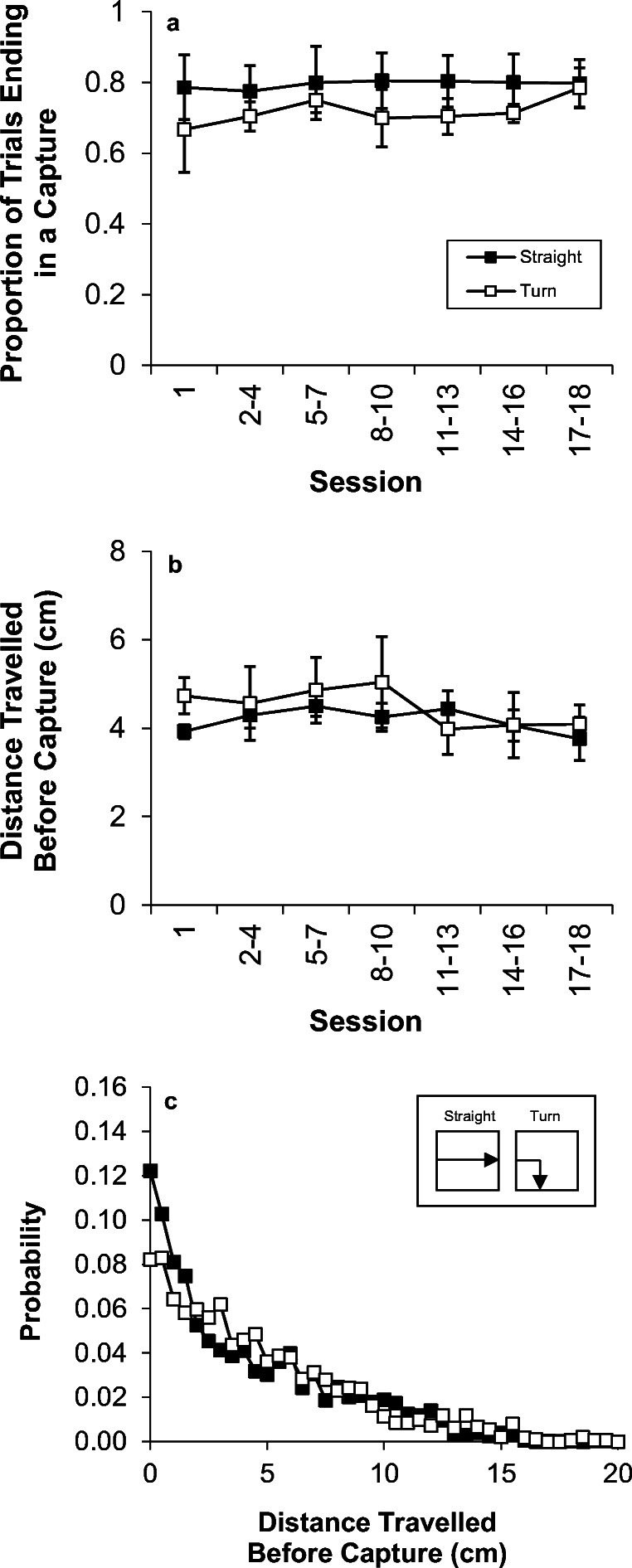
Results of Experiment 1. (a) The proportion of trials ending in capture response on straight and turn trials. (b) The mean distance travelled before capture on straight and turn trials. (c) The probability distribution of distances travelled before successful capture on each trial type

The two measures of capture responses are displayed in Fig. 1. The inset (panel c) presents a sketch of one of the possible motions for the straight and turn trial types. Panel a shows the proportion of trials that ended in a successful capture for the straight and turn trials as a function of blocks of sessions. As seen in the figure, capture success was approximately 80% on straight trials and the capture rate on turn trials was slightly lower. The capture rate did not appreciably change over the course of testing. An ANOVA with the variables of turn and session revealed no significant effect of turn on capture success, *F*(1,2) = 1.2. There also was no significant effect of session, *F*(6,12) < 1, nor was there any interaction between Turn × Session, *F*(6,12) < 1. In examining the individual birds, two of the birds performed slightly worse on turn trials and one bird performed similarly on both trial types.

Panel b displays the mean distance travelled from the start of the motion on straight trials or the turning point on turn trials until the point of capture. There was no indication of any effect of the turn, *F*(1,2) < 1, but there was an effect of session, *F*(6,12) = 3.1, *p* = .047, but no significant effect of Turn × Session, *F*(6,12) = 2.4, *p* = .091. In examining the individual birds, one bird captured the turn stimulus after a comparable distance to the straight trials, one bird was slightly later in capturing the stimulus on turn trials, and one bird was slightly earlier on turn trials. Overall, there was no consistent pattern to the performance across individuals. Panel c displays the distribution of capture locations. The greatest likelihood of capture occurred at the first point of eligibility and the distribution of capture locations did not appear to differ on straight and turn trials. The capture distributions of the individual birds were highly similar in shape to one another.

#### Errors

**Fig. 2.**
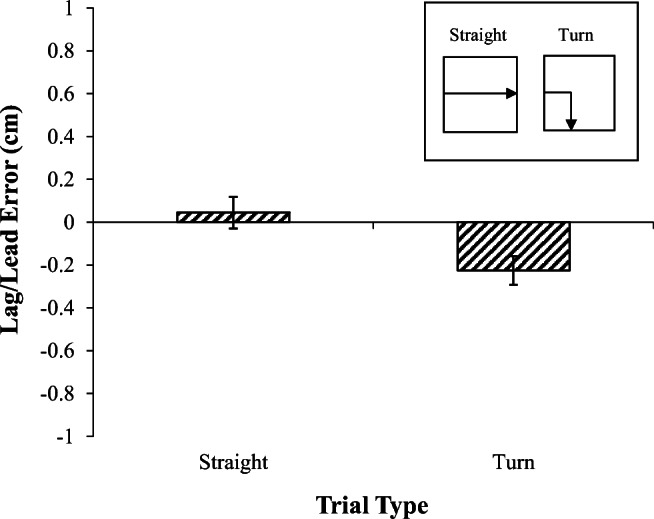
Error results of Experiment 1. Mean lag-lead error for each trial type. Inset shows an example of the motion on each trial type

Figure 2 displays the mean error location for both the straight and turn trials. Mean error values above zero reflect predominately leading errors, and mean error values below zero indicate lagging errors. On the straight trials the birds’ errors were slightly leading, whereas on the turn trials there was a tendency to lag behind the center of the stimulus. However, this apparent difference did not reach significance t(2) = 2.1.

To further pinpoint the effects of the sudden turn on errors, a regression analysis was conducted on the mean error positions (see *Data Analysis*) for each motion type following the turn (or, on straight trials, from the beginning of the trial) with the predictors of previous motion direction, current motion direction, and straight versus turn motion type. The dependent variable was the mean error relative to the center of the stimulus (averaged across both x and y coordinates. This revealed that the current motion was a significant predictor of error position, β = -.136, *t* = -2.13, *p* < .05, while the previous motion did not predict errors β = 0.11, *t* < 1, and straight versus turn did not differentially predict error positions, *t* < 1.

### Discussion

The introduction of a sudden unpredictable 90^o^ turn in the path of the moving stimulus had only a modest non-significant effect on the birds’ capture success, and there was no effect of the turn on capture location. This suggests that the birds are relatively adaptable in their capture ability. These findings differ from those of von Hofsten et al. (1998) with human infants. The infants were unable to adapt their reach after the turn, even when turn trials were presented in blocks. One difference may be that the change in direction occurred when the infants’ reaches were well underway. Given the speed and frequency of the pigeon peck, this was less likely to cause a problem. The findings also contrast with those of Lea et al. (2019) who found that pigeons tracking stimuli appearing in an arc trajectory were able to learn to adapt to unexpected changes in trajectory. However, the nature of the experiment was very different to that of our work.

Analysis of the errors also revealed an interesting pattern of results. The introduction of a sudden turn only had a subtle effect on mean error location, causing the birds to lag slightly behind the stimulus. Thus, adding an unpredictable element to the motion led to a slight loss of anticipation; however, this difference was not significant. Furthermore, the regression analysis revealed that the current motion direction was the only significant predictor of errors, indicating that the birds successfully adjusted their tracking to match the post-turn motion. This pattern of results differs from human infants, who are unable to override initial visual input from the beginning of each trial (von Hofsten et al., 1998). The difference between pigeons and infants may be due to differences in motor control which is not yet fully developed in human infants.

It is also worth noting that the use of constrained capture on test trials did not appear to disrupt performance. Additional analyses of the turn trials (not reported) revealed highly similar pecking patterns on the initial component of the trial in comparison to normal straight trials. And, there was no significant interaction of turn and session, indicating that the birds adapted fairly quickly to the turn stimulus. It therefore seems that the constraint did not impinge on the pigeons’ usual tracking performance.

The ability to adjust readily to the turn may have been promoted by the constant visual input available on the trial. Experiment 2 tested this possibility by inserting trials where there was a brief disappearance of the stimulus that accompanied the turn.

## Experiment 2

Research with rhesus macaques (*Maccaca mulatta*) found that they were able to anticipate the reappearance of a moving stimulus when it disappeared behind an occluder, but if it just disappeared (without apparent reason) they were unable to maintain pursuit over the disappearance (Churchland, Chou, & Lisberger, 2003). This suggests that the unpredictability of the disappearance caused the monkeys to lose their anticipatory tracking capabilities. If the pigeons are similarly affected, then we would expect to see a disruption in tracking following sudden disappearance on both straight and turn trials.

In the first phase, the pigeons were tested with disappearance trials where the stimulus suddenly and unexpectedly disappeared for 1.5 cm of the trajectory. Following reappearance, the stimulus could continue along the original trajectory or could reappear at a 90° angle to the original trajectory. A second phase used a 4.5 cm disappearance to examine whether the size of disappearance determined the magnitude of effects on capture and error responding. Disappearance trials were intermixed with normal straight and turn trials.

### Method

#### Animals

The birds from Experiment 1 participated in Experiment 2.

#### Apparatus

The apparatus was the same as in Experiment 1.

#### Procedure

##### Testing, Phase 1

Phase 1 of testing began immediately after the completion of Experiment 1. All three birds maintained above-criterion performance throughout the turn test and therefore required no further training. The birds received three types of test trials: 16 turn, 8 straight with disappearance, and 8 turn with disappearance. The turn trials were delivered in the same fashion as in Experiment 1.

The straight with disappearance trials involved a normal linear motion but the stimulus disappeared for 0.44 s, during which time it moved forward by 1.5 cm. Upon returning to the screen, the stimulus continued along its normal trajectory. On these trials, the pigeon could not capture the stimulus until after reappearance. The stimulus travelled for a minimum of 7.5 cm and a maximum of 15.0 cm prior to disappearance. The timing and location of reappearance was in accordance with the normal speed and trajectory of the stimulus. There were two straight-disappearance trials for each of the four directions of motion in each session.

The turn with disappearance trials were delivered in the same fashion as the normal turn trials, except that the stimulus disappeared for 0.44 s (1.5 cm) at the point of the turn. Thus, the stimulus reappeared on a different trajectory from the initial motion that was displaced by 90°, but at the correct time given a speed of 3.40 cm/s. There was one turn-disappearance trial for each of the eight possible motion combinations in each session.

There were a total of 32 test trials that were randomly intermixed with 40 normal training trials for a total of 72 trials. The contingencies of reinforcement were the same during the latter portion of the test trials (after the turn/disappearance) as for the normal training trials. The birds were tested until they met a criterion of at least 70% correct to each of the test types for two consecutive days; this required 40 sessions for Birds B50 and G83 and 50 sessions for Bird V42.

##### Testing, Phase 2

Phase 2 was conducted immediately after Phase 1 and was identical in all respects except that the period of disappearance was increased to 1.32 s (or a movement of 4.5 cm). The birds were trained to the same criterion as Phase 1, requiring 40, 22, and 30 sessions for Birds B50, V42, and G83, respectively.

### Results

#### Phase 1 Capture Responses

**Fig. 3.**
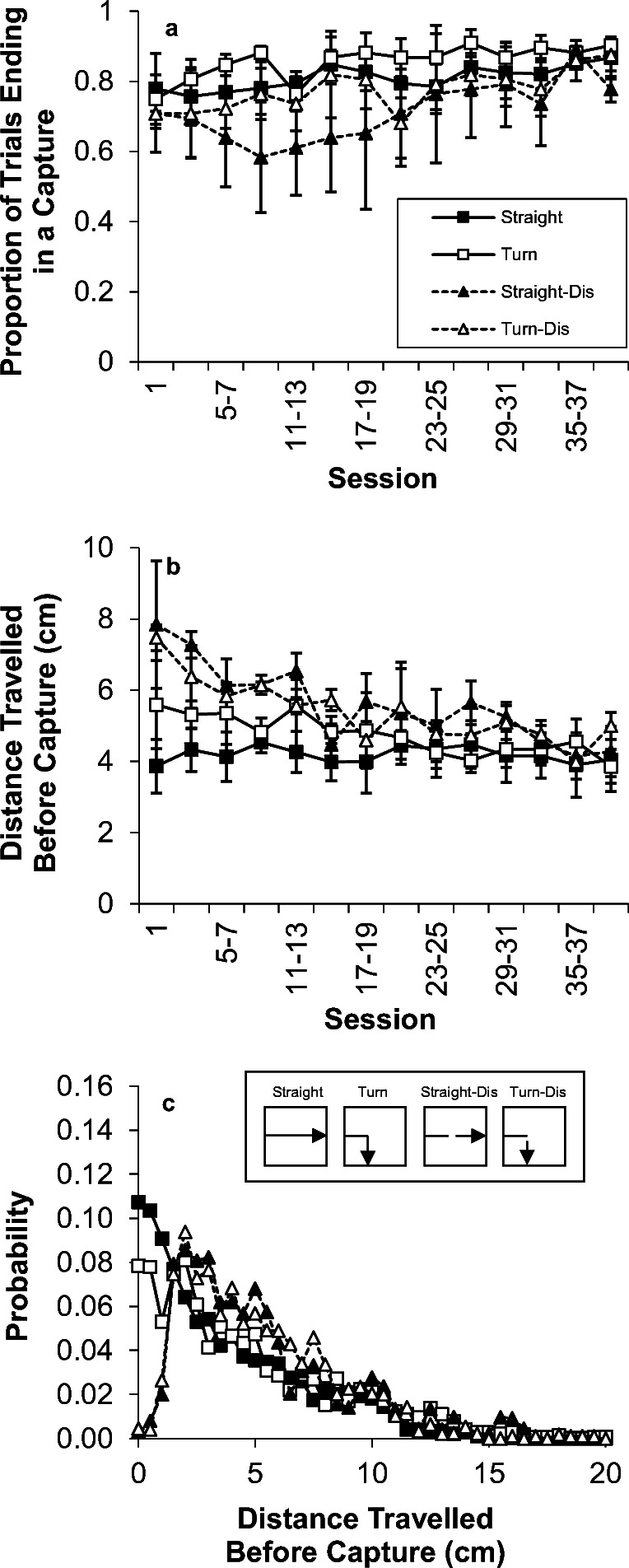
Capture results from Experiment 2, Phase 1. (a) The proportion of trials ending in capture response for the four trial types. (b) The mean distance travelled before capture for each trial type. (c) The probability distribution of distances travelled before successful capture on each trial type

Figure 3 displays capture responding as a function of trial type over the course of the test phase with straight and turn trials that were either smooth or had a disappearance for 1.5 cm of their path. A sample drawing of each trial type is displayed in the inset (panel e). Panel a shows the proportion of trials ending in a capture, for the normal training (straight), turn, straight with disappearance, and turn with disappearance trials. The straight and turn capture results were corrected for opportunity to capture (see Experiment 1, *Data Analysis*). As seen in the figure (panel a), it appears that the disappearance trials, particularly the straight-disappearance trials, resulted in poorer performance than the normal trials, a result that was verified by the statistical analysis, *F*(1,2) = 73.3, *p* < .05. This effect was driven heavily by Bird V42, which displayed a large disruption in performance following stimulus disappearance, particularly on the Straight-Dis trials. The other birds performed more similarly to all four trial types. There was no effect of the turn on capture success, *F*(1,2) = 1.1, no effect of session, *F*(13,26) < 1, Turn × Disappearance, *F*(1,2) < 1, Disappearance × Session, F(13,25) < 1, Turn × Session, *F*(13,26) < 1, or Disappearance × Turn × Session, *F*(13,26) = 1.2.

The mean distance travelled before capture is shown in panel b. Here, it was disappearance appeared to result in later capture, but this did not achieve statistical significance, *F*(1,2) = 12.4, *p* = .072. There was also an effect of session on performance, *F*(13,26) = 6.1, *p* < .001, due to improvements in performance over the course of testing. Although performance appeared to improve more for the disappearance and turn trials than for the straight trials, there was no interaction of session with the other variables [all Fs < 1.4]. Given that there were changes over sessions, further comparisons were conducted to assess the effect of disappearance on the mean capture location. Disappearance did result in significantly later capture in block 2, 4, 6, and 11 [smallest *t*(2) = 4.4, all *p*s < .05]. The effect of the disappearance on capture location can also be seen in the distribution of capture locations, shown in panel c. Stimulus disappearance resulted in a shift in the distribution so that peak in capture did not occur until around 1.5-2.0 cm after the stimulus reappeared; thereafter the capture distribution declined in a manner similar to the normal straight and turn trials. All three birds displayed a deficit in capture location following disappearance, but the magnitude of the effect varied somewhat across birds (B50: 1.5-2.0 cm; V42: 2.5-3.0 cm; G83: 1.0-1.5 cm).

#### Phase 1 Errors

**Fig. 4.**
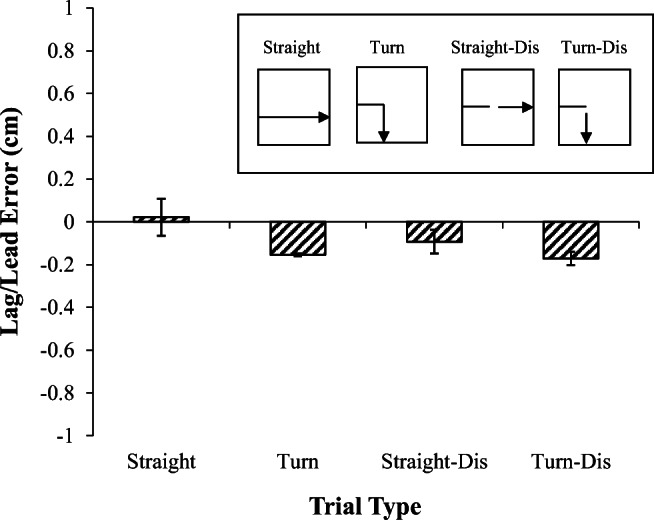
Error results of Experiment 2, Phase 1. Mean lag-lead error for each trial type. Inset shows an example of the motion on each trial type

Figure 4 displays the mean lag/lead error as a function of trial type. The mean error position on the straight trials was slightly ahead of the stimulus, whereas on the turn trials the birds showed more of a propensity to lag behind the stimulus, however the effect of turn on the mean lag/lead error did not reach significance, *F*(1,2) = 14.1, *p* = .064. The birds also appeared to lag more on the trials in which the stimulus disappeared briefly, however, again there was no significant effect of disappearance, *F*(1,2) < 1, nor was there any interaction of turn and disappearance, *F*(1,2) < 1.

A regression on the mean error locations with the predictors of previous motion, current motion, straight versus turn, and disappearance versus constant revealed a similar effect to Experiment 1 in that the current motion was the only significant predictor of the errors β = -.18, *t* = 4.79, *p* < .001. There were no significant effects of the previous motion, β = .04, *t* = 1.2, straight versus turn, β = .01, *t* = .16, or disappearance, β= -.06, *t* = -.72, on the error locations.

#### Phase 2 Capture Responses

**Fig. 5:**
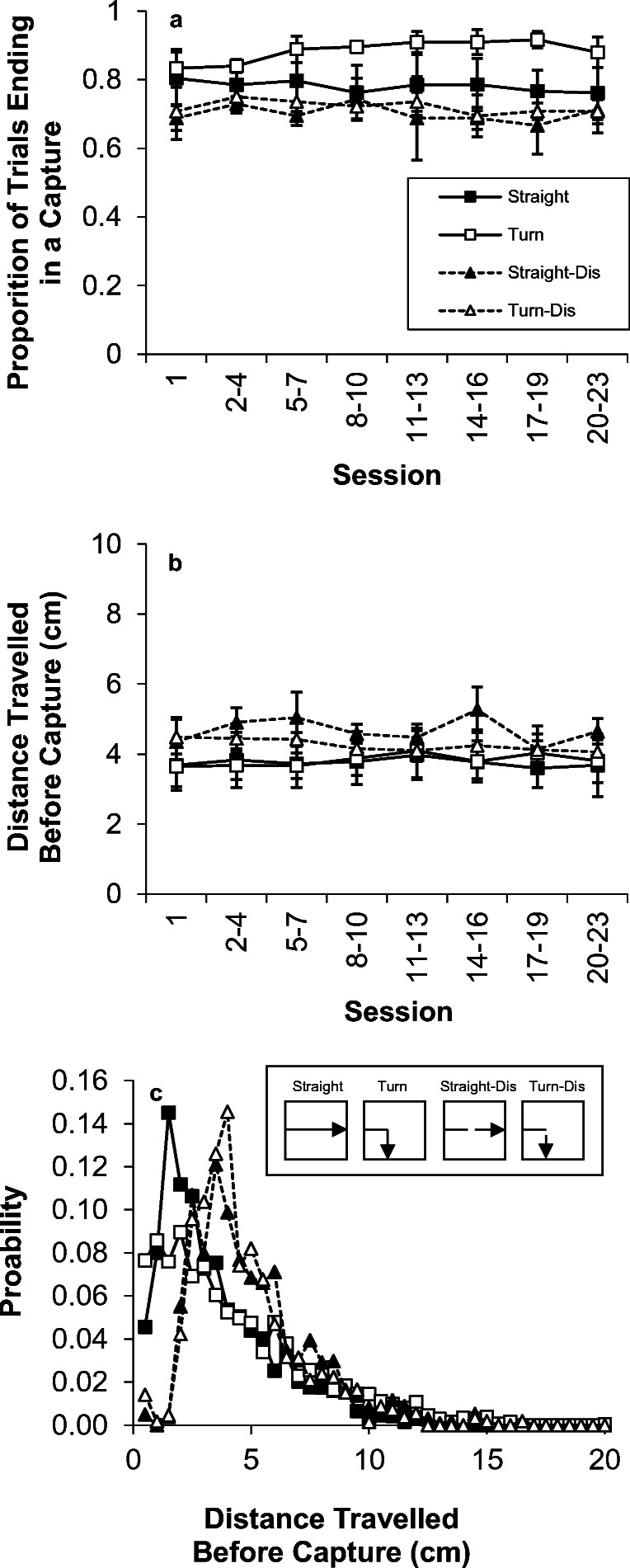
Capture results of Experiment 2, Phase 2. (a) The proportion of trials ending in capture response for the four trial types. (b) The mean distance travelled before capture for each trial type. (c) The probability distribution of distances travelled before successful capture on each trial type

Panel a of Fig. 5 displays capture success as a function of trial type over the course of Phase 2, where the stimulus disappeared for 4.5 cm of its path. There was no significant disruption in capture success on disappearance trials, *F*(1,2) = 6.3, *p* = .127. There was no effect of turn, *F*(1,2) = 2.1, nor was there any Disappearance × Turn interaction, *F*(1,2) = 1.8. There also was no effect of session, nor any interaction of session with the other variables, all *Fs* < 1. In examining the individual birds, all three birds displayed a modest deficit in capturing the Straight-Dis and Turn-Dis stimuli, and one bird exhibited an additional deficit in capturing Turn stimuli. The magnitude of the deficits varied across birds from 10%-30%.

Panel b reveals an effect of disappearance on mean capture location, *F*(1,2) = 26.6, *p* = .036, where the stimulus was caught later on disappearance trials. There was no effect of the turn, *F*(1,2) < 1, or Turn × Disappearance interaction, *F*(1,2) = 5.1. There also was no change over sessions, *F*(7,14) < 1, nor was there any interaction of session with any of the other variables (largest *F* = 1.2). The distribution of capture locations (panel c) shows that the peak in capture responses occurred approximately 2.5-3 cm later and then declined gradually. All three birds displayed later capture on disappearance trials (both Straight-Dis and Turn-Dis), but the magnitude varied slightly across birds (B50: 2.0-3.0 cm; V42: 2.5-3.0 cm; G83: 2.5-3.5 cm).

#### Phase 2 Errors

**Fig. 6.**
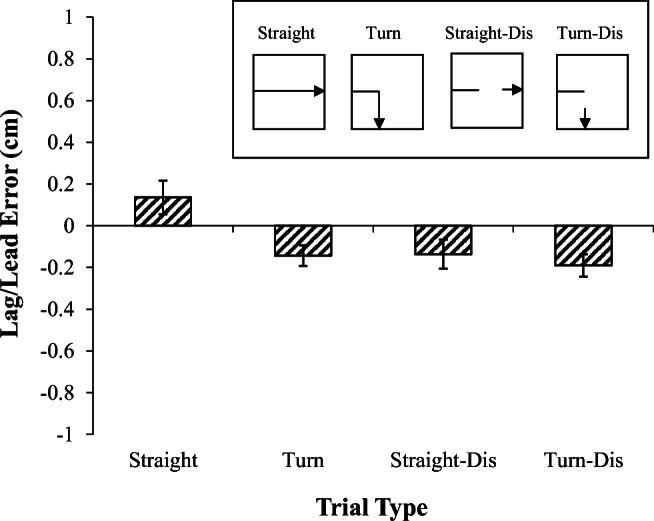
Error results from Experiment 2, Phase 2. Mean lag-lead error for each trial type. Inset shows an example of the motion on each trial type

Figure 6 displays the mean error as a function of trial type during the test phase with straight and turn trials with and without disappearance for 4.5 cm of its path. The mean error position on the straight trials was slightly ahead of the stimulus, whereas on the turn and on both the disappearance trials the birds appeared to lag slightly behind the stimulus, an ANOVA revealed a significant effect of turn, *F*(1,2) = 43.5, *p* < .05. There was also a main effect of disappearance, *F*(1,2) = 83.7, *p* < .01, and a significant Turn × Disappearance interaction, *F*(1,2) = 82.3, *p* < .01. Post-hoc analyses revealed that the interaction was due to the birds pecking significantly ahead of the stimulus on the straight normal trials compared to the other trial types.

A regression examining the role of the current and previous motion on errors on normal and disappearance straight and turn trials revealed the same pattern of results as in the previous phase, with a strong effect of the current motion direction on error locations, β = -.17, *t* = -4.70, *p* < .001. There was no effect of the previous motion, β = .037, *t* = 1.0, straight versus turn, β = 0.01, *t* = .02, or disappearance, β = -.05, *t* = -.62.

### Discussion

The disappearance of the stimulus for either 1.5 cm (0.44 s) or 4.5 cm (1.32 s) had only a modest, non-significant effect on capture success, indicating that the disappearance did not disrupt capture. However, both durations of disappearance caused a decrease in capture efficiency and thus the stimulus was caught later on the disappearance trials. In fact, it appeared that the probability of capture at a given point shifted approximately 1.5 cm later for the trials with a 1.5-cm disappearance and 3 cm later for trials with a 4.5-cm disappearance. This suggests that some disruption to the anticipatory mechanism occurred and that longer periods of disappearance were more disruptive.

This idea is supported by the error analyses. The short disappearance had a moderate (albeit non-significant) effect on the mean error position in that the birds lagged behind the stimulus more. When the stimulus disappeared for a greater period (4.5 cm), there was a greater effect on the mean error position with an increase in lagging errors. Regression analyses revealed that the current motion was the only significant predictor of error locations, indicating that the birds were correctly tracking the post-turn motion, even if they were lagging to a greater extent. If there had been a residual effect of the pre-turn motion on performance, then this would have indicated some persistent effect of the early visual information on pecking locations, but this was not the case.

Overall, the pattern of results in the present study is consistent with the notion that the disappearance resulted in a period where the pigeons disengaged from tracking the stimulus. When the stimulus reappeared, there was a lag before tracking was re-engaged and this was greater than the normal lag to begin tracking at the start of a trial. This was seen both in the later capture and greater lagging errors, and the effect was larger when the stimulus disappeared for a longer period. But, once tracking was re-engaged, the birds appeared to track the motion appropriately.

Studies with humans (Bennett & Barnes, 2003, 2004) and rhesus macaques *(Maccaca mulatta*; Churchland et al., 2003) suggest that smooth eye pursuit (and therefore anticipation) cannot be maintained in the unexplained absence of a visual target. These effects are consistent with the present observations with the pigeons. The effects of disappearance may have been due, at least in part, to a loss of attention to the stimulus. The sudden disappearance mimics the end of a trial following a capture response (but without reinforcement), so the pigeons may have engaged in post-trial behaviors during the disappearance. Longer disappearances would be more likely to engage these behaviors.

In smooth pursuit tasks, disappearance of a stimulus behind an occluder results in both monkeys and humans successfully anticipating the point of reappearance. The sustained ability to track a stimulus behind an occluder may be due to the presence of a salient visual cue, which allows for prediction of possible positions of reappearance. If so, then we would expect the pigeons to behave as expecting reappearance on a straight trajectory and thus would experience disruptions in adjusting to a turn that occurs behind the occluder. This was tested in Experiment 3.

## Experiment 3

Occlusion of part of the path of a moving stimulus has frequently been used to assess the role of prediction in tracking in infants (Gredebaak et al., 2002; von Hofsten et al., 2000). Rosander and von Hofsten (2004) established that at only 3 months of age, infants were able to form representations that persisted over 300 ms of occlusion. However, infants under the age of 5 months were unable to correctly predict changes in velocity that occurred behind an occluder.

Using both linear motion and sudden turns, von Hofsten et al. (2000) found that 6-month old infants did not naturally extrapolate motion over an occluder; however they rapidly learned to extrapolate on linear trials. On the non-linear (turn) trials, they were also able to learn to anticipate the motion, however this took much longer and the overall accuracy was lower. By the time infants reach 9 months of age they were able to make precise predictions about where an occluded object should reappear on a circular trajectory (Gredebaak et al., 2002). They showed no tendency to make linear extrapolations (which would result in a miss) but behaved as though the velocity, direction and motion would be the same behind the occluder as it was prior to disappearance. The results indicate that performance was not based solely on inertial properties, but on a non-linear extrapolation process. Adults tested on the same task produced similar results (Gredebaak et al., 2002).

Adult rhesus macaques were able to anticipate the reappearance of a stimulus moving along a linear path when it disappeared behind an occluder (Churchland et al., 2003). However, if the stimulus suddenly disappeared they were unable to maintain pursuit over the period of disappearance. In Experiment 2, there were some similarities in performance between our pigeons and Churchland et al.’s monkeys. The disappearances caused a delay in capture and a propensity to lag behind the stimulus. However, the regression analyses revealed that the pigeons could re-engage and appropriately track the post-turn, post-disappearance motion. The delay in capture and lagging errors implies that the birds did not maintain a representation of the motion after the stimulus disappeared. An occluder, however, may produce very different results. Not only does it more closely approximate disappearances in real world situations, it also provides a visual cue which could allow predictions of where the stimulus would reappear. This may allow the anticipatory component of tracking to survive a period of stimulus absence.

### Method

#### Animals

The birds from Experiments 1 and 2 participated in Experiment 3. Due to building work in the laboratory, they were rested for a period of 3 months. During this time, they were housed in a separate part of the facility under normal housing conditions and were maintained at 90-95% of their free feeding weights. In the two weeks prior to initiation of the experiment, their weights were gradually returned to 85-90% through restricted feeding.

#### Apparatus

The apparatus was the same as in Experiment 1, except that the experimental programs were written in MatLab (version 7.1). The switch in software was necessary because the addition of an occluder produced undesirable effects on the speed and smoothness of motion in E-prime.

#### Training Procedure

Due in part to the transfer to MatLab for experimental programming and in part to the long rest period between experiments; we conducted two phases of retraining. There was an initial 15 days with the training regimen used in Experiment 1 with the experimental program that had been generated in E-prime. We then followed this immediately with 22 days of training with the Experiment 1 training protocol written in MatLab. The data from the two training phases was checked for deviations that could have been due to changes in the experimental software. Performance in the initial retraining stage with E-prime was comparable to performance on training trials in Experiments 1 and 2. Under the MatLab program, there were no qualitative differences in performance, but the birds did show some minor improvements in capture success and increases in anticipatory errors, but these data did not differ significantly from the E-prime training results.

#### Testing Procedure

Following the training phase, the birds received tests with three types of trials: 16 with turns, 8 straight with occluder, and 8 turn with occluder. The occluder was a 1.5 x 1.5 cm gray square that appeared on the screen on all trials for the entire duration of the trial. On straight occluder and turn with occluder trials, the occluder was positioned along the path of motion so that the stimulus appeared to move behind the occluder and then re-emerge from behind it. On straight trials, the stimulus emerged straight across from where it had disappeared and on turn trials, the stimulus emerged in a position that was 90° displaced from the point of occlusion. The position of the occluder matched the 1.5-cm disappearance condition in Experiment 2, and in all other respects these trials were identical to the disappearance trials in Phase 1 of Experiment 2.

The normal straight training trials and normal turn trials also contained an occluder, but it was displaced away from the path so that on these trials the stimulus never came into contact with the occluder. The on-screen position of the occluder on these trials was matched with the position on the occluder test trials, but the stimulus passed near the occluder instead of moving behind it. The contingencies of reinforcement were the same as in Experiment 2, with constrained capture on both turn and occluder test trials. On the turn trials, the stimulus could not be caught until after the turn and on occluder tests the stimulus could not be caught until after it emerged from behind the occluder.

### Results

#### Capture Responses

**Fig. 7.**
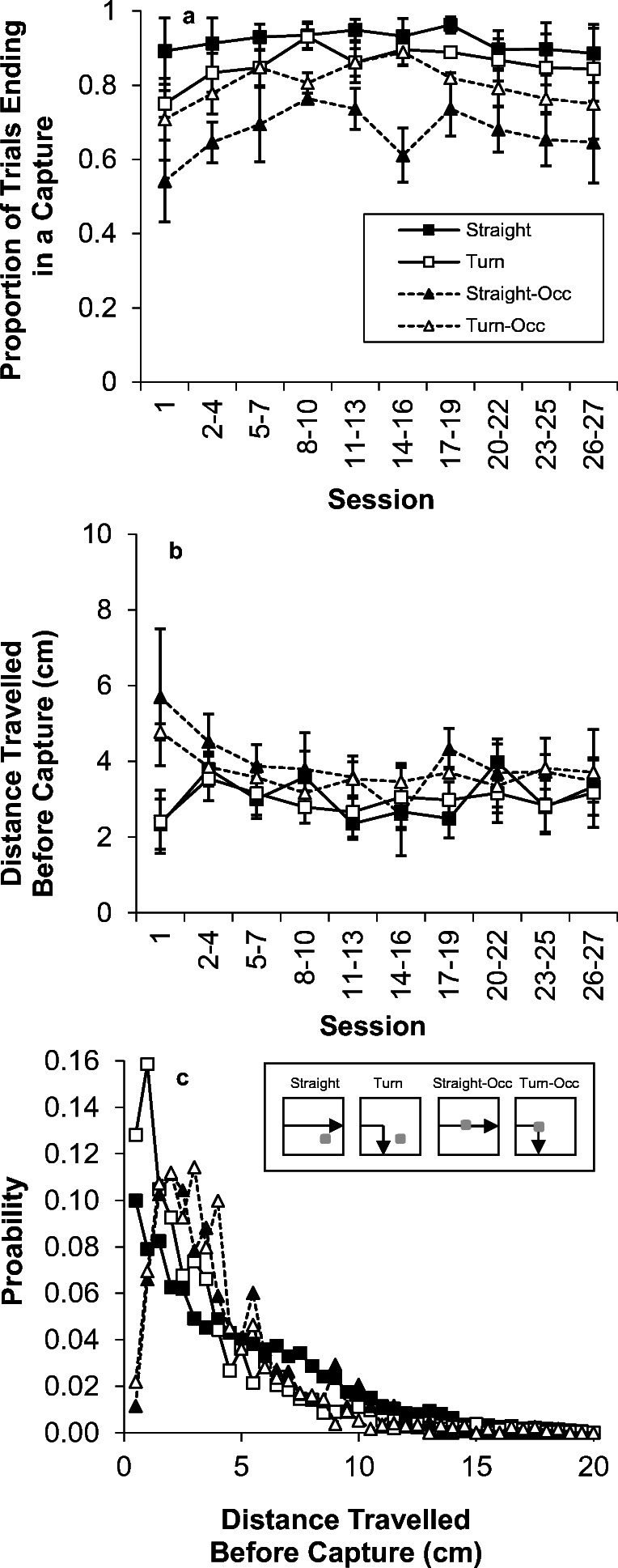
Capture results from Experiment 3. . (a) The proportion of trials ending in capture response for the four trial types. (b) The mean distance travelled before capture for each trial type. (c) The probability distribution of distances travelled before successful capture on each trial type

Capture responses are displayed in Fig. 7. The proportion of trials ending in a capture (panel a) was disrupted by occlusion, *F*(1,2) = 21.9, *p* = .043. There was no effect of the turn on capture success, *F*(1,2) = 4.6, but there was an Occlusion × Turn interaction, *F*(1,2) = 230.1, *p* = .004. Follow-up analyses indicated that indicated that the Straight-Occ trials resulted in poorer performance than the Straight and Turn-Occ trials. There was no effect of session, *F*(9,18) = 1.3, nor any interaction of session with the other variables (largest *F* = 1.4).

There also was a significant main effect of occlusion on the mean capture location, shown in panel b, *F*(1,2) = 19.2, *p* = .048. However, there was no effect of turn, session, or any interactions of turn or session with the other variables (largest *F* = 1.9). In examining the distributions of capture locations (panel c), it appears that occlusion resulted in a modest shift in the peak capture location of around 0.5-1.0 cm. The individual birds all displayed later capture on the occlusion trials, but the magnitude of the effect varied across birds (B50: 0.5-1.0 cm; V42: 1.0-1.5 cm; G83: 1.0-1.5cm).

#### Errors

**Fig. 8.**
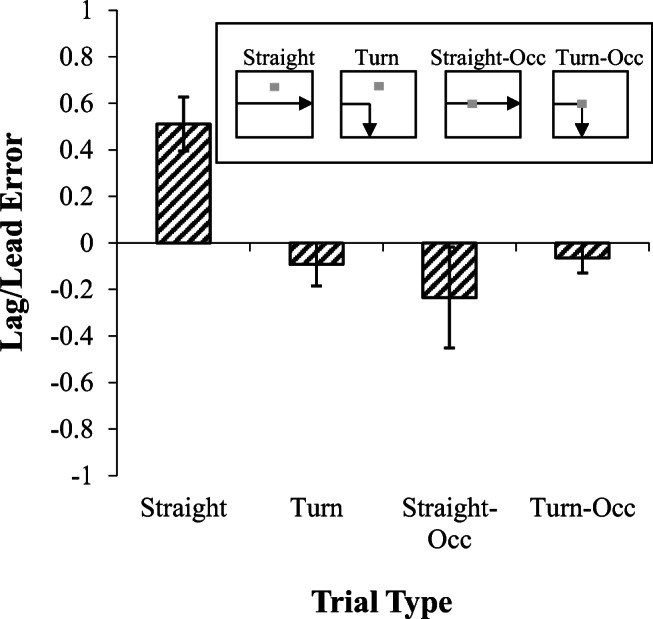
Error results from Experiment 3. Mean lag-lead error for each trial type. Inset shows an example of the motion on each trial type

Figure 8 displays the mean error as a function of trial type during the test phase with straight and turn occluded and normal trials. The mean error position on the straight trials was in front of the stimulus, whereas on the turn and on the occlusion trials the birds lagged behind the stimulus. An ANOVA revealed no effect of turn *F*(1,2) = 5.7, but did reveal a main effect of occlusion, *F*(1,2) = 48.3, *p* < .05, with birds lagging more on the occluder trials. There was a also a significant Turn × Occlusion interaction, *F*(1,2) = 29.7, *p* <. 05. Post-hoc analyses revealed that the significant interaction was due to the birds pecking significantly further ahead of the stimulus on the straight normal trials compared to the other motions.

A regression examining the role of the previous and current motion on post-occlusion errors on normal and occluded straight and turn trials revealed that previous motion direction was the primary predictor of error locations, β = 0.09, *t* = 2.0, *p* < .05. The current motion direction did not predict errors β = 0.02, *t* = .5, and there was once again no overall impact of straight versus turn, β = .03, *t* = .3, but there was a significant effect of occlusion versus non-occlusion on errors, β = -.19, *t* = -2.0, *p* < .05.

### Discussion

A 1.5-cm occlusion of the moving stimulus disrupted capture success on straight-occluder trials. This stands in contrast to the general lack of effect that turns and disappearances had on capture success in the previous experiments. A disruption of capture success indicates a loss of stimulus control over tracking behavior, and that there was a general disruption in the tracking process. It is possible that the presence of a novel stimulus on the screen may have caused this disruption by competing with the moving stimulus for attention, as has been observed in infants (Rosander & Von Hofsten, 2004). Occlusion also resulted in later capture on both Straight-Occ and Turn-Occ trials, displacing capture by around 0.5-1.0 cm. This indicates that the birds did not fully anticipate the timing of reappearance of the stimulus from behind the occluder. However, the magnitude of the delay in capture was smaller in this study compared to the effect of disappearance on capture location suggesting that the birds may have partially anticipated reappearance of the stimulus.

Disruption of tracking was also seen in the error analysis. Occlusion caused the birds to peck primarily behind the stimulus when it re-emerged, suggesting that there was some loss of the anticipatory process. In addition, the regression analysis revealed that the previous motion significantly predicted errors, suggesting that the birds anticipated that the stimulus would emerge directly on the opposite side of the occluder from where it disappeared. When the stimulus turned during the occlusion, the errors remained consistent with the previous motion. For example, if the stimulus was moving rightward but turned to move upward during occlusion, then the error positions following the turn/occlusion were predicted by the initial rightward motion. In other words, the errors were consistent with errors to a rightward motion even though the stimulus was now moving upwards.

The pattern of the occlusion results is consistent with elements of predictive tracking in monkeys and humans in that the pigeons anticipated the stimulus reappearance on the other side of the occluder. This produced a substantial disruption in performance when the stimulus turned while occluded. The pigeons also showed a similar pattern to human infants in that the presence of an occluder produced a general disruption in capture performance, at least on a subset of the trials. Overall, the pigeons predicted where the stimulus should reappear, but their lagging errors indicate that they did not predict when the stimulus should reappear.

## General Discussion

When the birds were presented with sudden turns in motion without any disappearance or occlusion there was little effect on capture success or capture location, and the effect on errors was to produce a modest (non-significant) increase in lagging errors. The regression analysis conducted on the errors indicated that the birds successfully adjusted their tracking and used the correct motion following unpredictable turns when visual input was available during the turn (Experiment 1). However, disappearance resulted in a temporary loss of tracking behavior with a delay to re-engage tracking following reappearance (Experiment 2). It seems that once tracking was re-initiated following disappearance, then the pattern of errors was consistent with the post-disappearance motion regardless of whether the stimulus turned.

It is surprising that the disappearance in the visual input did not simply reset the pigeon tracking mechanism. The pigeons’ tracking lagged behind the stimulus significantly more after reappearance compared to tracking from the start of the trial. This suggests that the lack of predictability disrupts anticipatory tracking because the position of disappearance was less predictable than the potential position of trial onset. This idea is supported by previous findings. Wilkinson and Kirkpatrick (2011) showed that intermixed presentation of two highly predictable motions that were originally presented in blocks resulted in a loss of anticipation. This is unexpected given that the only difference was between-trial predictability. The findings of Churchland et al. (2003) also support the idea that unpredictability of stimulus disappearance caused the monkeys to lose their anticipatory tracking.

Occlusion produced a different pattern of results. The presence of another stimulus on the screen resulted in some general disruption in capture accuracy. This may have been due to the birds’ paying attention to the occluder instead of tracking the stimulus. The disappearance behind an occluder lowered capture success and delayed capture. The regression analysis indicated error patterns that reflected the initial motion suggesting that the birds were anticipating a linear motion following occlusion. This resulted in delayed capture and lagging errors on the turn-occlusion trials, both of which are indicators of poor anticipation.

One critical difference between the occluder and the disappearance trials is that the presence of an occluder provides a cue for stimulus disappearance and an end point of that disappearance. It is possible that if the disappearances presented in Experiment 2 were cued in some manner (a change in stimulus color, or presentation of a tone) then the pigeons (and other species) may be able to learn to represent the motion during disappearance.

Disappearance behind an occluder is also more ecologically relevant. Objects rarely disappear in nature, but they do become occluded by other objects. This is particularly important during predator-prey interactions. To be able to maintain a representation of a moving object (including the direction and speed of motion) over an occlusion is highly adaptive for both predator and prey animals. Despite this clear adaptive value, pigeons are notoriously poor at recognizing partially occluded objects (e.g., Cerella, 1980; Ushitani & Fujita, 2005; Ushitani, Fujita, & Yamanaka, 2001). However, pigeons are able to learn to recognize partially-occluded stimuli under some circumstances (Kirkpatrick, Wilkinson, & Johnston, 2007; Lazareva, Wasserman, & Biederman, 2007). The pigeons in this study were given no occlusion training, though, the presence of motion may allow them to access this ability more readily.

The use of touchscreen technology allowed us to monitor both capture and error behavior with great accuracy. However, resistive touchscreens do not count every single peck because some lighter intensity pecks fall below the resistance threshold. As such, some information is likely to be missed and this could affect the results, for example, by reducing capture rates. However, given that this issue is present on all trials we do not believe it impacts our overall interpretation of the results. Another issue relates to how pigeons peck when confronted with smaller objects as used here. When smaller grain-like stimuli are presented, pigeons peck in a manner consistent with how they peck at food (Jenkins & Moore, 1973). Specifically, the beak tends to open as if to grab the stimulus. As such, the pigeon could potentially touch the screen with either the lower or upper part of its beak. When two touches occur simultaneously, the screen would record the strongest touch rather than recording both touches. It would be interesting to develop technology that monitors the position of both the lower and upper beak during tracking to give a more comprehensive picture. While our method allowed us to test the birds when moving in a natural manner, the touchscreen only records the final output of tracking - the peck. This contrasts with much of the human and primate literature in which eye and hand movement are also measured. It would therefore be interesting, in future work, to assess head or eye movements prior to the peck.

Wilkinson and Kirkpatrick (2009) found that pigeons appear to anticipate the object position by aiming their peck a constant spatial amount ahead of the stimulus, rather than a constant temporal amount as reported in human infants. Two factors are involved in tracking and capture behavior in the pigeon (Wilkinson & Kirkpatrick, 2009). Lag time appears to be an inevitable result of the mechanics of the pigeon peck. This alone would cause errors to lag the stimulus. The second factor, anticipatory bias is more complex. It allows the pigeons to predict a fixed spatial amount ahead of the stimulus regardless of the velocity of motion, but only when the motion is highly predictable. The anticipatory bias is highly influenced by prior motion history, both long-term (Wilkinson & Kirkpatrick, 2009) and short-term (Wilkinson & Kirkpatrick, 2010). In contrast, humans appear to possess a sensorimotor system for anticipatory tracking, the predictive extrapolation mechanism (Von Hofsten, 1987). Predictive extrapolation involves the use of previous velocity and trajectory information to predict the future location of an object. As a result, humans anticipate a constant temporal amount ahead of the stimulus of approximately 200 ms. A temporal bias is more adaptable than a spatial bias because temporal bias allows for accurate anticipatory tracking even when the velocity changes. However, a temporal bias would require more sophisticated computations of the bias parameter under conditions of changing velocity. It would be interesting to test a wider range of species to determine if the temporal bias is a feature of predatory species (vs. prey), mammals in general (vs. birds), or is specific to primates or indeed only present in humans.

Despite the apparent differences in the mechanisms underlying their tracking behavior, the pigeons’ responses to disappearances and occlusions appear to be similar to those observed in primates (e.g. von Hofsten et al. 2000, human infants; Churchland et al. 2003, rhesus macaques). This suggests that the role of visual input in tracking behavior may have similar effects across species. Further experiments in which the target disappearance time is systematically varied would provide insight into the nature of the internal representation of motion and may elucidate the similarities and differences in tracking across species.
